# A landscape-scale assessment of tropical mammals reveals the effects of habitat and anthropogenic disturbance on community occupancy

**DOI:** 10.1371/journal.pone.0215682

**Published:** 2019-04-19

**Authors:** Nathalie Cavada, Rasmus Worsøe Havmøller, Nikolaj Scharff, Francesco Rovero

**Affiliations:** 1 Tropical Biodiversity Section, MUSE—Museo delle Scienze, Trento, Italy; 2 Center for Macroecology, Evolution and Climate, Natural History Museum of Denmark, University of Copenhagen, Copenhagen, Denmark; 3 Section for Evolutionary Genomics, Natural History Museum of Denmark, Copenhagen, Denmark; 4 Department of Anthropology, University California, Davis, Davis, California, United States of America; 5 Udzungwa Ecological Monitoring Centre, Udzungwa Mountains National Park, Mang’ula, Tanzania; 6 Dipartimento di Biologia, Università degli Studi di Firenze, Sesto Fiorentino, Italy; Cornell University, UNITED STATES

## Abstract

With biodiversity facing unparalleled threats from anthropogenic disturbance, knowledge on the occurrences of species and communities provides for an effective and fast approach to assess their status and vulnerability. Disturbance is most prominent at the landscape-level, for example through habitat loss from large-scale resource extraction or agriculture. However, addressing species responses to habitat changes at the landscape-scale can be difficult and cost-ineffective, hence studies are mostly conducted at single areas or habitat patches. Moreover, there is a relative lack of studies on communities, as opposed to focal species, despite the former may carry more comprehensive information. Here, we used a multi-region, multi-species hierarchical occupancy model to study a meta-community of mammals detected by camera traps across five distinct areas within a heterogeneous landscape in Tanzania, and aimed to assess responses to human disturbance and environmental variables. Estimated species richness did not vary significantly across different areas, even though these held broadly different habitats. Moreover, we found remarkable consistency in the positive effect of distance to human settlements, a proxy for anthropogenic disturbance, on community occupancy. The positive effect of body size and the positive effect of proximity to rivers on community occupancy were also shared by communities. Results yield conservation relevance because: (1) the among-communities consistency in responses to anthropogenic disturbance, despite the heterogeneity in sampled habitats, indicates that conservation plans designed at the landscape-scale may represent a comprehensive and cost-efficient approach; (2) the consistency in responses to environmental factors suggests that multi-species models are a powerful method to study ecological patterns at the landscape-level.

## Introduction

Addressing the current unparalleled loss of biodiversity requires an understanding of what shapes ecological communities, especially those exposed to anthropogenic threats, how they vary in space and time, and what are the implications of such variation [1–3]. This is because determining vulnerability of communities likely yields greater potential for informing conservation than single species or guilds [4]. Community level approaches not only provide inference on individual species, but they also address patterns at the ecosystem level, which is an ecologically- and cost-effective approach for management actions [5]. Moreover, even if management actions might be designed for a limited set of target species, management likely exerts an influence that extends to other species within a landscape [6]. In this context, tropical forest mammals are of central relevance: they are among the most threatened taxa on earth and yet they carry out fundamental ecosystem functions as seed consumers and dispersers, predators, and prey [7]. Changes in mammalian communities are therefore likely to have consequences for ecosystem stability [8]. Moreover, the larger-bodied species tend to require large undisturbed areas and are particularly impacted by uncontrolled threats such as logging, hunting and environmental degradation [9–11], to which they are variably sensitive [12,13]. Previous research on anthropogenic effects on mammal communities has had a marked focus on variation of species richness [1,14] and abundance of individual species [3,15]. Results from these studies generally point to a decline in both metrics across gradients from intact versus degraded, modified and/or hunted habitats. Relative less attention, however, has been devoted to metrics that describe the overall abundance and distribution of communities, such as occupancy [6]. This is important because average community occupancy may provide for a finer resolution assessment of community responses to anthropogenic disturbance and environmental change than species richness [15]. This, in turn, may provide for an informative tool in conservation management [6].

Hierarchical multi-species occupancy models have proven to be effective methods to address spatial patterns of communities, as their hierarchical structure integrates information from multiple species [16,17]. In addition, they account for species-specific imperfect detection by processing data that are replicated in space and time, which are typically generated from camera trap surveys. Such methods exploit collective data on the community and provide information on occurrence probabilities for both the observed and unobserved species, hence including those that are rare and elusive, for which inference can be highly improved [6,18]. Importantly, multi-species models can also test the influence of habitat and human disturbance factors on the richness and distribution of species in the community. Besides targeting communities, the spatial scale at which analyses are performed is also of relevance, as anthropogenic disturbance often impacts biodiversity at larger scales than that of the single forest or habitat patch [19]. This is especially true within complex human-natural systems, where effects at multiple spatial scales, i.e., from site- to landscape-level, drive species occurrences [20,21]. Thus, adopting a broader perspective appears critical, yet without overlooking effects that can arise at the local scale [22–24]. Both contexts, landscape and local, are essential to understand trends in species and communities and to efficiently prioritize conservation strategies and manage threatened populations [25]. However, addressing responses of multiple species to habitat and management with a landscape perspective often requires expensive and time consuming sampling and analysis [26]. This is also why results are typically derived for single communities and comparisons are made post-hoc, among independently-studied communities in each region of interest [27–30].

Here, we address the importance of community-level and of multi-area occupancy analysis to study a meta-community of medium-to-large mammals in Tanzania. We used the recently developed multi-region occupancy model [31] to analyse camera trapping data collected in distinct areas within the heterogeneous landscape of the Udzungwa Mountains of Tanzania, a renowned hotspot of biodiversity in the Eastern Arc Mountains [32]. Several studies based on camera trap surveys have been conducted in the area [10,33–35], some of which addressed community patterns in single areas of the present study. These studies have raised concern about the protection of mammals that undergo severe pressures originating from both hunting and habitat degradation [36]. Here, we collected a dataset in the Udzungwa Mountains which is unprecedented in terms of both spatial extent and sampling effort. Our specific objective was to examine spatial patterns in the occurrence of the mammal meta-community at the landscape-level to (a) assess variation in estimated species richness of the communities (meant as area-specific assemblages) occurring across the landscape; (b) determine how body size of species, and environmental and anthropogenic factors, influence both meta-community and species occupancy; (c) evaluate if examining patterns at the landscape-scale provides for a conservation-relevant approach. Given the study area is completely surrounded by human settlements, and the known implications in terms of disturbance to biodiversity within protected areas [37], our main expectation was that anthropogenic disturbance consistently impacts meta-community occupancy, despite differences in the species composition of each community and the main habitat type where they occur.

## Materials and methods

### Study area and species

**Fig 1:**
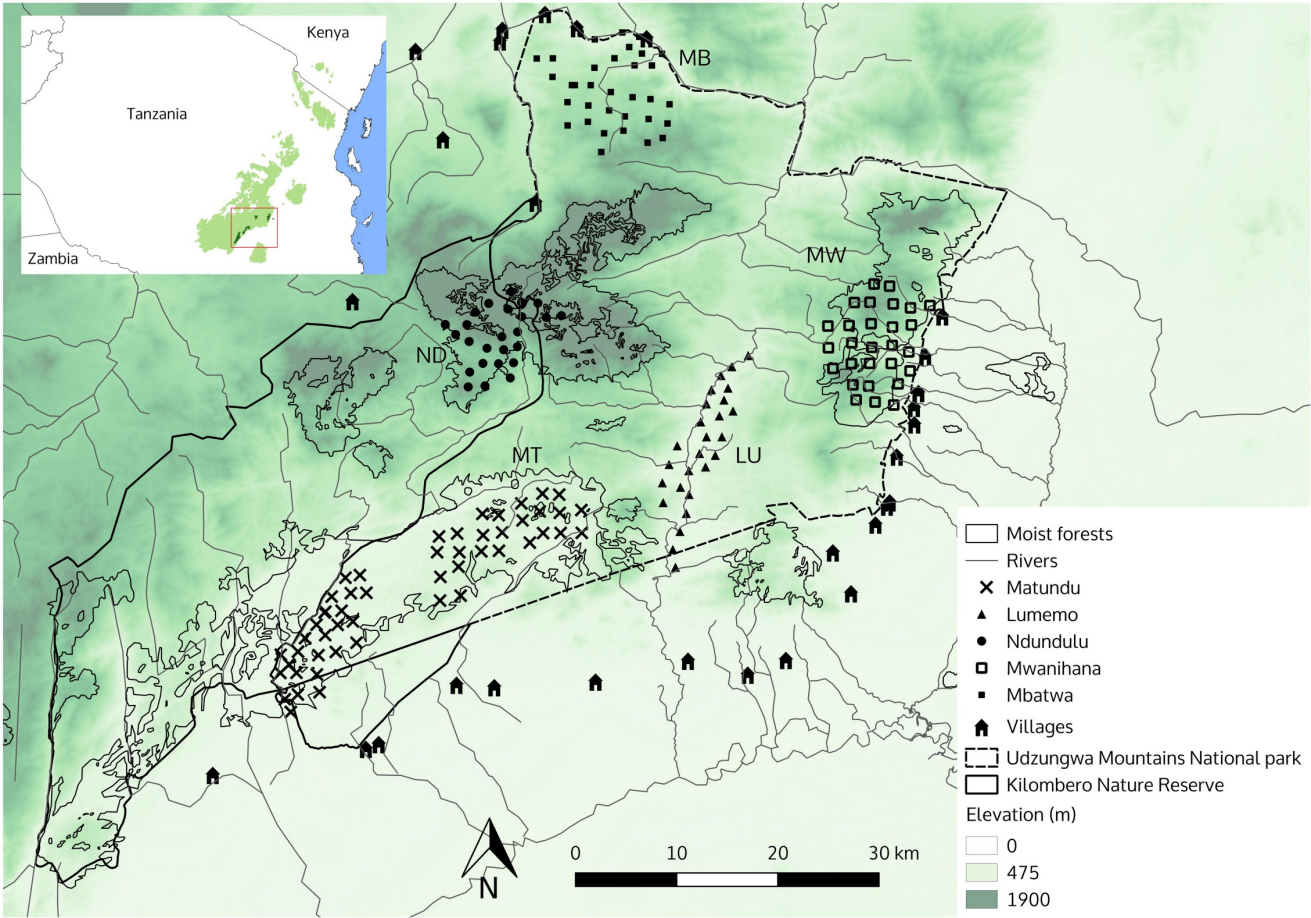
Study area. Map of the Udzungwa Mountains of Tanzania, showing the National Park, the Kilombero Nature Reserve and the location of six camera trap arrays placed in five different areas (MT = Matundu, MB = Mbatwa, LU = Lumemo, ND = Ndundulu, MW = Mwanihana) characterised by different habitat types (Table 1). The background layer is a Digital Elevation Model, with darker colour indicating higher elevation.

The Udzungwa Mountains, located in south-central Tanzania, are part of the Eastern Arc Mountains, a renowned biodiversity hotspot [34,38], and represent an outstanding region for mammalian richness and endemism in East Africa [35]. The area is a mosaic of closed forest blocks interspersed with drier habitats, from dense dry forest to woodland and wooded grassland [39]. While such heterogeneity of habitats is largely natural, shaped by climate and terrain morphology [39], the sharp and degraded edges of closed forest blocks are likely the result of anthropogenic disturbance, especially near the protected area boundaries. Indeed the area is surrounded by subsistence farming to the north, west and south and by sugar cane intensive farming to the east; these surrounding areas are densely punctuated by human settlements. The Udzungwa Mountains National Park (UMNP; 1,990 km^2^) and adjacent Kilombero Nature Reserve (1,345 km^2^) form a united portion of protected areas separated from the Selous Game Reserve to the east and Uzungwa Scarp Nature Reserve to the southwest ([34]; Fig 1). For the placement of camera trap arrays we identified five geographically distinct areas that broadly include the major variation in habitat types in this large protected area (Table 1); these consist of the largest closed forest blocks as well as the intervening, drier habitats, as follows: (1) Lowland Afrotropical rainforest in the southern UMNP (ranging from 280–800 m a.s.l.), Matundu (MT); (2) dry *Acacia-Commiphora* woodlands in the northern UMNP, Mbatwa (MB), surrounded by dry baobab woodlands at low elevation and by grasslands at high elevation (500–1900 m); (3) *Brachystegia* woodlands in the central valleys of UMNP (300–800 m), Lumemo (LU); (4) Afromontane forest in Ndundulu (ND), west of UMNP and part of the Kilombero Nature Reserve (1200–2300 m); (5) Rainforest escarpment of Mwanihana (MW), in the eastern part of UMNP (280–2100 m). Target species of our analyses were medium-to-large, predominantly ground dwelling mammals that are detected by camera traps. These species consist of both specialists, including a number of narrow range and endemic species (mainly found in the montane forest blocks) and more generalist, wider ranging species found across multiple areas (this study; [35]).

**Table 1:** Survey effort and areas. Survey effort for the five areas in the Udzungwa Mountains, Tanzania, sampled with camera traps. These areas hold broadly different habitat types, as indicated in table; the elevation range (in m a.s.l.) is related to sampling sites.

Area	No. of camera trap sites	Survey period	Dominant habitat (elevation range)
Matundu	51	Sep.—Dec. 2013	Lowland, regenerating forest (284–675)
Mbatwa	34	Jun.—Jul. 2014	Dry dense forest to wooded grassland (508–1804)
Lumemo	26	Jul.—Sep. 2014	Riverine forest and woodland (336–736)
Ndundulu	25	Sep.—Oct. 2014	Montane moist forest (1196–2285)
Mwanihana	28	Oct.—Dec. 2014	Forest escarpment, from lowland deciduous to montane moist forest (284–1799)

### Data collection: Camera trapping

Data from camera traps come from a previous study on leopard density by RWH and followed the protocol designed for leopard studies in African forests [40]. A regular grid was placed over the study area with trap sites spaced by 1.6 km. Six camera trap arrays, each of 25–51 camera trap stations covering 738 km^2^ were placed in the five areas described above (Fig 1). Camera trap arrays were sequentially sampled in the dry season, which is defined as months with < 100 mm average rainfall [7], i.e., from August to December 2013 and from June to December 2014, and each camera trap operated 31 days on average (range 12–49 days; Table 1). Each camera trap station consisted of two cameras placed at 30–40 cm above the ground level, facing each other and at a distance of 3–4 m from the centre of an animal trail. In 87 of 164 sites, at each station, a white Xenon flash Cuddeback Ambush camera (Cuddeback Non Typical Inc., USA) was paired with one infrared camera UOVision 565HD IR+ (UOVision Technology, Shenzhen, China) set on 15-second video recording mode. Remaining stations consisted of pairs of Cuddeback Ambush cameras. Camera trapping realized an effort of 31.13 camera days on average from 164 sites that were effectively sampled.

### Data collection: Covariates and species traits

We hypothesized average community occupancy and detectability of mammal communities across the landscape to be mainly influenced by a set of three habitat and anthropogenic disturbance covariates (Table 2). (1) We considered distance to human settlements a proxy of anthropogenic disturbance, that increases with proximity of camera trap sites to villages. Villages are mainly localized immediately outside the reserve boundaries (Fig 1). This is where human encroachment can more likely occur also due to the presence of roads and agricultural activities [34]. Predictably, we found this covariate to be correlated to distance from protected area boundaries (Pearson's r = 0.65), indicating that it also accounts for the extent of protected habitat. (2) We used distance to the nearest river, to evaluate the effect of the proximity to water sources, that we hypothesized could positively affect the occupancy of mammals [41,42]. Data for both these covariates came from digitized topographic maps by the Surveys and Mapping Division, Government of Tanzania, and were calculated using the function ‘v.distance’ in GRASS software [43]. Lastly, we included (3) vegetation cover, to evaluate the effect of habitat type. To quantify vegetation cover we used a land cover map based on ground-truthed Landsat data [44]. We calculated the percentage cover of vegetation types within a buffer with radius 500 m around each camera trap site. We found that the most represented vegetation types were moist evergreen forest (mean of 48%), *Brachystegia* woodland and mixed woodland with grassland and shrubs (22%), grassland (11%), and bushland (6%). Moist forest cover correlated negatively with the other three categories lumped (Pearson's *r* = -0.82), therefore we only used this category as the dominant habitat type and as indicator of drier and open vegetation types. In addition to these covariates we also used species body mass (log-transformed; data sourced from [45]) as key species trait, that we assumed to affect both detectability and occupancy.

**Table 2:** Habitat and anthropogenic covariates and species traits. Covariates and traits used to model community occupancy (*ψ*) and detectability (*p*) across the landscape, with predictions for their effect on these parameters (see text for further details).

Covariate	Abbreviation and unit	Source	Prediction of effect
Distance to settlements	dSettl (m)	Digitized 1:10,000 topographic maps	Positive on *ψ* and *p*: both parameters increases away from direct anthropogenic disturbance
Distance to rivers	dRiv (m)	Digitized 1:10,000 topographic maps	Negative on *ψ*: occupancy decreases away from permanent rivers
Forest cover	forCover (%)	Landsat imagery	Positive on *ψ*: occupancy increases with forest cover
Body mass	mass (kg)	Smith et al. [44]	Positive on *ψ*: larger species have wider distribution and are better detected; negative on *p*: larger species are more elusive and their homer range overlaps less frequently with camera trap sites

### Statistical analysis

We analysed camera trap data by using a multi-species occupancy model [17] that was recently extended to a multi-region formulation [31,46]. This allowed us to jointly estimate the ecological metrics of interest across populations and communities occurring in the five distinct areas. Thus, three spatial scales were involved in our model: (1) ‘sites’, where camera traps recorded species occurrences, (2) collection of sites in each ‘area’ (or ‘region’), where we estimated species richness of each community, as well as the effects of covariates and body mass on occupancy and detectability of each species, and (3) the ‘landscape’ encompassing the five regions and hosting the meta-community, for which we modelled the average occupancy and detectability and how covariates and body mass influenced these parameters.

We implemented a Bayesian community occupancy model, using data augmentation to estimate richness in each area while accounting for the number of unobserved species [16]. The procedure of data augmentation assumes a Uniform(0, *M*_*r*_) prior for *N*_*r*_, the ‘true’ number of species in each sampled region *r* = 1, …, *5*, where *M*_*r*_ is arbitrarily defined, equal across regions (i.e. *M*_*r*_ = *M*), and larger than the total number of species detected in the largest community (i.e., *M* >> max(*n*_*r*_)). As suggested by Kéry and Royle [47], we fixed *M*_*r*_ to 100, i.e., approximately three times max(*n*_*r*_). Following Sutherland et al. [31], we organized the multispecies encounter frequency data from the five regions in a 3-dimensional array *Y*, with elements *y*_*kir*_ where *k* = 1,…, *n*_*r*_ being the species sampled at sites *i* and region *r*, so that *y*_*ir*_ ≥ 1 if the species *k* was detected at site *i*, and *y*_*ir*_ = 0 if it was not detected; we then augmented the arrays with *M*—*n*_*r*_ species with all-zero encounter histories (*y*_*ir*_ = 0). Within the data augmentation framework, the random sampling of the *N* occurring species from the total of *M* potential species in each area was modelled as *w*_*kr*_ ~ Bernoulli(*Ω*_*r*_), with *Ω*_*r*_ being the data augmentation parameter, and *w*_*kr*_ being the species-specific indicator variable, denoting whether species *k* was present in the *r*^th^ community (*w*_*kr*_ = 1) or whether it was a structural zero (*w*_*kr*_ = 0 and therefore z_*kr*_ = 0). For species that were detected in a region, we fixed *w*_*kr*_ = 1 [31]. We note that the data augmentation parameter and *N*_*r*_ are equivalent parameters, since the expected value of *N*_*r*_ is *M*_*r*_*Ω*_*r*_. Thus, *Ωr* essentially represents the proportion of *M*—*n*_*r*_ ‘all-zero’ encounter histories representing undetected species members of the *r*^*th*^ community.

The state process (i.e. the occurrence of species across sites in each region) was modelled as *z*_*kir*_|*w*_*kr*_ ~ Bernoulli(*w*_*kr*_*ψ*_*kir*_), where z_kir_ = 1 for an occupied site and z_kir_ = 0 for an unoccupied site by species *k*, with *ψ*_*kir*_ representing the species-specific occurrence probability. We described the observation process (i.e. detection) as *y*_*kir*_|*z*_*kir*_ ~ Bernoulli(*z*_*kir*_*p*_*kir*_), with *p*_*kir*_ representing the detection probability. To investigate the response of species occurrence to the set of covariates, we regressed occupancy and detection probabilities of species across sites and in each region on both site covariates and body mass as a species trait, using a logit-link function:

$$logit(\psi_{kir})={\beta 0}_{kr}+\beta 1\times {logmass}_{kr}+{\beta 2}_{kr}\times {dRiv}_{ir}+{\beta 3}_{kr}\times {dSettl}_{ir}+{\beta 4}_{kr}\times {forCover}_{ir}$$

$$logit(p_{kir})={\alpha 0}_{kr}+\alpha 1\times {logmass}_{kr}+{\alpha 2}_{kr}\times {dSettl}_{ir}$$

where 'logmass' indicates the log-transformed mass, 'dRiv' the distance to the nearest river, 'dSettl' the distance to human settlements, and 'forCover' the moist forest cover. We then modelled intercepts and slopes of both regressions as species-specific random effects, drawn from a common prior distribution for the community and with mean and variance hyper-parameters representing the average intercept and slopes across the observed mammal community and the variation among species, respectively. Importantly moreover, given we were interested in estimating average occupancy and detectability of the whole meta-community, we assumed the intercepts to be constant across regions, i.e. we constrained them to a common mean and variance:

$${\beta 0}_{kr}∼Normal(\mu_{\beta 0},\sigma_{\beta 0}^{2})$$

$${\alpha 0}_{kr}∼Normal(\mu_{\alpha 0},\sigma_{\alpha 0}^{2}).$$

We applied the same procedure to the slopes, such as, for example:

$${\beta 1}_{kr}∼Normal(\mu_{\beta 1},\sigma_{\beta 1}^{2}).$$

Lastly, we considered the species trait mass for each community to be drawn from a normal distribution: mass_*kr*_ ~ Normal(*μ*_*massr*_, *σ*^*2*^_*massr*_) with *μ*_*massr*_ and *σ*^*2*^_*massr*_ representing the average mass and the average variance of species in region *r*. This allowed us to estimate the average community mass in each sampled region.

We implemented the models in a Bayesian framework using JAGS (version 4.2.0; [48]) via R (version 3.2.3; [49]) with the R2jags (version 0.5.7–7; [50]) package. We generated three parallel chains of 50,000 iterations with a burn-in of 5,000 iterations and thinning by 10 for a total of 13,500 draws that were used to derive summaries of parameter posterior distribution. We specified a Normal(0, 0.01) prior distribution on the logit scale for the data augmentation parameter (*Ω*_*r*_), and for mean community detection (*μ*_*α0*_) and occupancy (*μ*_*β0*_) parameters. We specified a Uniform(-10, 10) prior distribution for all the covariate coefficients on detection and occupancy (*α1*, *α2*_*kr*_, *β1*, *β2*_*kr*_, *β3*_*kr*_, *β4*_*kr*_). Convergence of the Markov chains was satisfactory based on the Gelman-Rubin statistic, which was always ≤1.04 [51]. We evaluated covariate effects by considering whether 95% Bayesian credible intervals (BCIs) encompassed zero. The model code is reported in S1 Appendix and the .RData file for the analysis is available at https://doi.org/10.6084/m9.figshare.7892843.

## Results

Camera traps yielded 3725 events per day of 48 species of medium-to-large, ground-dwelling mammals: 29 species in Matundu, 33 in Mbatwa, 24 in Lumemo, 25 in Ndundulu and 24 in Mwanihana (see S1 Table for the checklist of species by taxonomic order and body mass). Only 13 of 48 species detected were shared among all study areas. These included large and wide-ranging, or common herbivores such as elephant (*Loxodonta africana*), African buffalo (*Syncerus caffer*), and bushbuck (*Tragelaphus scriptus*), small-bodied antelopes such the Harvey’s duiker (*Cephalophus harveyi*) and suni (*Neotragus moschatus*), the aardvark (*Orycteropus afer*), as well as both large (such as the leopard, *Panthera pardus*), and small (bushy-tailed mongoose, *Bdeogale crassicauda*) carnivores. Estimates of community size (median) at the region level ranged from 28 species in Lumemo to 37 species in Mbatwa, with 95% BCIs that overlapped among regions (Table 3).

**Table 3:** Area-specific data and model results for species richness and body mass. Summaries of species richness, estimated richness, body mass of species detected and average estimated body mass for communities of mammals sampled by camera traps in five areas in the Udzungwa Mountains of Tanzania.

Area	No. of species detected	Estimated species richness (median and 95% BCI)	Mean (range) of body mass (Kg) of species detected	Estimated average body mass (mean and 95% BCI, log scale)
Matundu	29	31 (29–37)	45.83 (1–465)	-0.06 (-0.57–0.41)
Mbatwa	33	37 (34–43)	25.70 (1–117)	0.17 (-0.21–0.53)
Lumemo	24	27 (24–32)	19.88 (1–68)	0.19 (-0.28–0.62)
Ndundulu	25	30 (26–36)	17.24 (1–73)	-0.10 (-0.61–0.35)
Mwanihana	24	29 (25–37)	26.67 (1–175)	-0.17 (-0.72–0.30)

**Fig 2:**
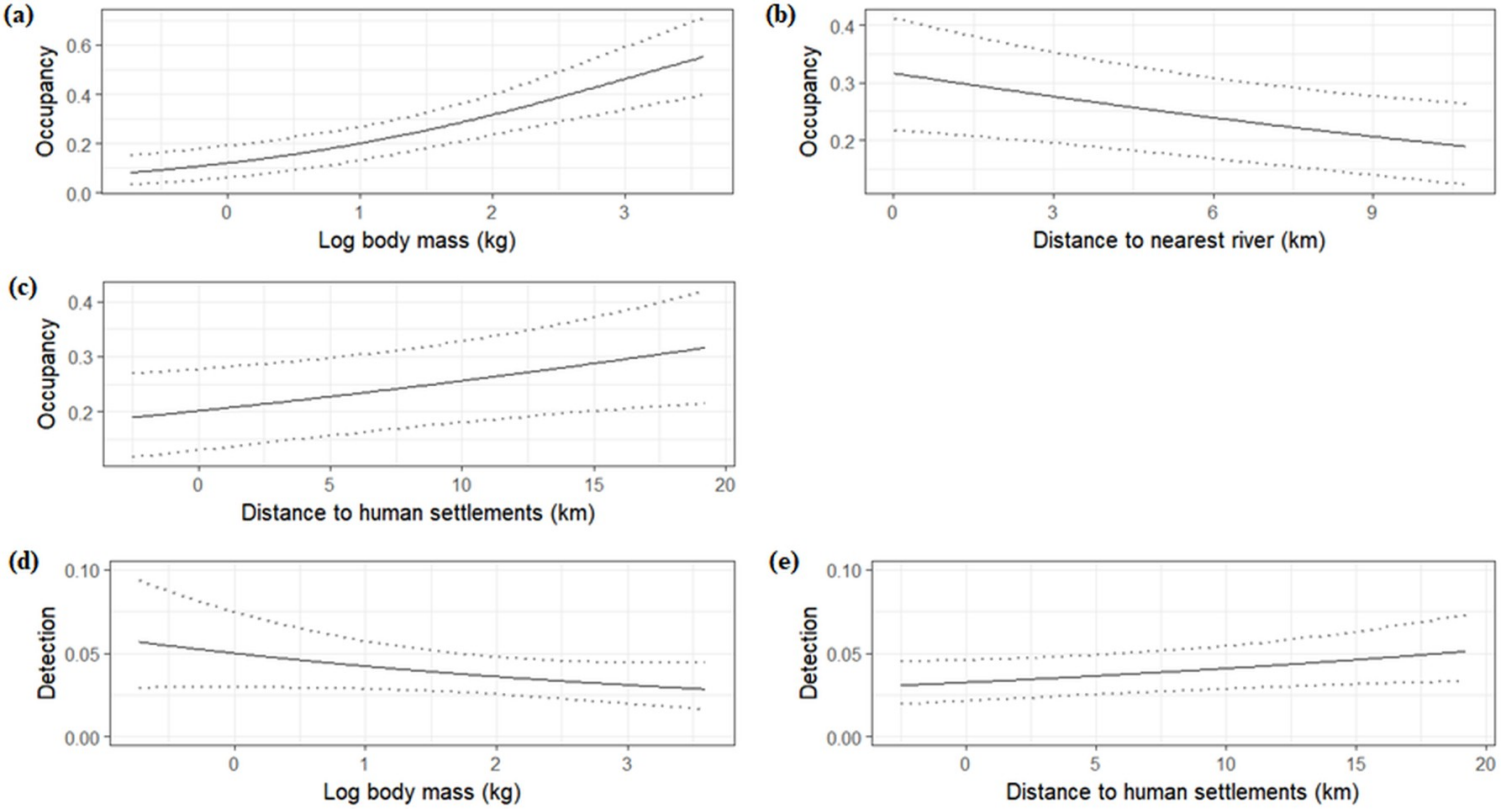
Covariate effects on occupancy and detection at the landscape scale. Mammal meta-community responses of occupancy probability to body mass (a), distance to the nearest river (b) and distance to human settlements (c), and of species detection probability to mass (d) and distance to human settlements (e) in the Udzungwa Mountains of Tanzania.

The average occupancy probability for the entire meta-community was 0.26 (95% BCI: 0.19–0.33) and the average detection probability was 0.04 (0.03–0.05). We found a negative, but not significant effect of body mass (*α1* = -0.14, -0.40–0.12, 84.4% probability) and a significant, positive effect of distance to human settlements on community detection probability (*α2* = 0.17, 0.02–0.33) (Table 4, Fig 2, S1 Fig). We also found community occupancy to be positively and significantly influenced by both species mass (*β1* = 0.76, 0.43–1.13), and distance to settlements (*β3* = 0.23, 0.06–0.41). We found instead a negative and significant effect of distance to rivers (i.e. a positive effect of proximity to rivers) on occupancy, (*β2* = -0.20, -0.34 –-0.06) (Table 4, Fig 2, S1 Fig). We did not find any effect of forest cover on community occupancy. The body mass of detected species ranged from 0.18 to 3,940 kg, and the mean mass of detected species in each community ranged from 185.37 kg in Ndundulu to 227.55 kg in Matundu (Table 3). The average estimated species mass in the meta-community, on the log scale, was lowest in Mwanihana (-0.17) and highest in Lumemo (0.19; Table 4).

**Table 4:** Posterior distributions from model results. Summaries of posterior distributions from the multi-region hierarchical occupancy model for mammal meta-community in the Udzungwa Mountains of Tanzania. Parameters *α* and *β* are given on the logit scale and are the coefficients for detection probability and occupancy, respectively.

Parameter	Mean (95% BCI)
*α1*	-0.14 (-0.40–0.12)
*α2*	0.17 (0.02–0.33)
*β1*	0.76 (0.43–1.13)
*β2*	-0.20 (-0.34 –-0.06)
*β3*	0.23 (0.06–0.41)
*β4*	-0.14 (-0.37–0.09)

*α1* and *β1* = parameters for species body mass (log-transformed); *α2* and *β3* = distance to human settlements; *β2* = distance to nearest river; *β4* = moist forest cover.

We also derived the species-specific effects of covariates on occupancy and detectability, to assess how species responses in each region varied in comparison to the meta-community parameter estimates. Despite variation among species, only for the effect of distance to human settlements on detection (*α2*) the variability among species was such that for a number of them (range 1–7 per community) the effects differed significantly from the average (i.e., their 95% BCIs did not overlap the average estimate and BCIs of the hyperparameter; S2 Fig). Interestingly, even though the species composition differed considerably among communities, there appeared to be consistent responses among species that occurred in multiple regions. In addition, while we designed the model to estimate covariate effects at the landscape scale (i.e., at meta-community level), the signs of species-specific effects for covariates that significantly affected meta-community occupancy were generally consistent among the five communities (S2 Fig). This suggests similarities between community and meta-community responses to covariates.

## Discussion

We studied species richness and community occupancy across five sampled areas in a highly heterogeneous landscape in Tanzania. We investigated how habitat variables and anthropogenic disturbance affect meta-community occupancy while also analysing species-specific effects, thereby conducting a comprehensive evaluation of mammalian occurrence patterns at the landscape scale. Despite the pronounced differences in community composition (only 27% of species occurred in all study areas), and the heterogeneity of habitats where they occur, we found shared patterns of responses by the whole meta-community to the drivers considered. In particular, we found that occupancy of the meta-community was positively related to the distance from human settlements with a significant relationship. We consider this covariate a proxy for decreasing human disturbance. The shared pattern of responses we found is in line with previous findings on generalized mammal responses to extent of protected habitat and disturbance across the Eastern Arc Mountains [10], and, more specifically, with similar findings from studies on the abundance of leopard [52] and of arboreal primates in the Udzungwa Mountains [37]. The latter study, in particular, found similar responses for the density of three species of arboreal primates to signs of human disturbance, with these signs being generally more abundant in forests more heavily surrounded by human settlements. These evidences altogether point to a marked impact of anthropogenic disturbance on species and communities at the landscape-level, and suggest that the mammal meta-community in the area is facing threats from anthropic settlements and activities regardless of the high variability in species composition and habitat type. While the increasing impact on mammals near anthropic areas and the positive effect of protected habitat are documented patterns [10,53,54], our finding of consistent responses at the meta-community level is noteworthy and of conservation relevance, as it suggests that effective conservation plans could be designed and coordinated across the landscape. On the contrary, current conservation efforts tend not to be coordinated, with different protected areas and authorities involved, and management strategies that are not harmonized across the landscape [37,55]. As a result, there are areas that are relatively well protected and areas that are not, regardless of their biodiversity importance. Therefore, coordinating protection efforts appears a strategy to increase the cost-effectiveness of law enforcement, as well as guide the design of comprehensive conservation measures such as buffer zones, wildlife management areas, and community-conservation schemes. Moreover, the among-species consistency in responses to covariates within communities supports the notion that species-specific conservation action plans (typically designed for endemic and/or highly threatened and charismatic species) will likely provide for benefits to the whole guild or community of mammals [6].

In addition to the consistent effect of distance to human settlements on meta-community occupancy, we found a number of other shared ecological patterns. The significant and positive effect of proximity to rivers, albeit weak, is noteworthy: the Udzungwa Mountains represents an important watershed at local and national levels, and the network of protected areas is fundamental to ensure its integrity by preventing alteration by surrounding human activities [21]. Meta-community occupancy was seemingly not influenced by forest cover, however we consider this a possible spurious result associated with the fact that within the large gradient in habitats that characterises the study area, moist forest was relatively less represented in our sample than drier habitats with more open cover such as woodland and grassland/bushland. Additionally, we found that the closed moist forests we sampled host relatively fewer and more specialized, forest-dwelling species than the communities in drier and more open habitats (see below), which likely determined the non-significance of forest cover. This finding mirrors results from a community occupancy study from Botswana [3] that found marked variability of mammals in the responses to vegetation cover, mainly associated with species trophic guild. We also found a consistent, positive effect of species mass on community occupancy, indicating that larger species tend to have a wider distribution and require larger areas than smaller species [56,57]. The positive effect of distance from human settlements also held for detection probability, indicating a general pattern of increasing elusiveness of animals nearer to disturbed areas, matching results from an earlier study in one of the target areas [34]. In contrast to the signal for occupancy, species body mass tended to negatively affect the probability of detection, however the effect was not significant. This result matches an earlier analysis on one of the target community [34]: authors suggest that this relationship may reflect behavioural responses, with larger species being less detected because of their greater elusiveness. Additionally, larger species likely have a relatively smaller portion of their home range falling into the detection zone of camera traps [58]. In this respect we note that for the larger-bodied and large-ranging species the camera trap site spacing design may violate the assumption of non-independence of detections among adjacent sites, therefore for these species the metric of occurrence is more likely site use than occupancy [16].

Our hierarchical model with data augmentation allowed us to estimate species richness while accounting for species that were potentially missed by camera traps. We highlight the importance of the data augmentation procedure we followed, that is not based on the known species list for the area (often referred to as the regional species pool [47]). For rich biodiversity areas such as the Udzungwa Mountains, accurate knowledge on occurring species is often lacking, as also indicated by the number of new records of species occurrence we reported (see below). The multi-region framework allowed us to take into account previous knowledge that most species did not occur in all study areas. We could therefore overcome the limitations of a joint analysis that treats species as if they belonged to a single community, which would confound absence with non-detection [31]. While we did not find significant differences in richness across areas, there appeared a signal of higher richness in the Mbatwa area (mixed dry woodland and wooded grassland), followed by Matundu (lowland forest). Along with the woodland at Lumemo area, Mbatwa also holds the community with relatively larger-bodied species. This evidence indicates that closed and moist forests support communities that are relatively less species-rich and are composed of smaller-bodied, specialist species [59]. These species are known to be more susceptible to fluctuations in environmental conditions [60,61]. Indeed the Udzungwa Mountains hold a number of endemic and range-restricted species, such as the Udzungwa red colobus (*Procolobus gordonorum*), Sanje mangabey (*Cercocebus sanjei*), grey-faced sengi (*Rhynchocyon udzungwensis*) and Abbott’s duiker (*Cephalophus spadix*), all of which are listed under the higher threat categories by the IUCN. In this regard, our comprehensive survey provided for a number of species records that further affirm the exceptional importance of the Udzungwa Mountains for mammalian diversity. Thus, our study yielded the first records of blue duiker (*Philantomba monticola*) in lowland rainforest (Matundu), and confirmed the presence of klipspringer (*Oreotragus oreotragus*), Sable antelope (*Hippotragus niger*), ground pangolin (*Smutsia temminckii*), caracal (*Caracal caracal*), serval (*Leptailurus serval*), Meller’s mongoose (*Rhynchogale melleri*) and Jackson’s mongoose (*Bdeogale jacksoni*). In contrast, a number of large mammals included in the earlier checklist [35] were not recorded by our camera traps, including the African wild dog (*Lycaon pictus*), aardwolf (*Proteles cristatus*), side-striped jackal (*Canis adustus*), eland (*Taurotragus oryx*) and lesser kudu (*Tragelaphus imberbis*).

In conclusion, our study suggests that a hierarchical modelling approach represents a useful tool to test ecological hypotheses of variation in community richness and abundance, allowing for a comprehensive assessment of responses to habitat and anthropogenic factors. We suggest this approach might also help optimize resources for field sampling and data analyses, especially in heterogeneous and/or fragmented landscapes [18,62]. Our findings on the patterns of responses to ecological and anthropogenic factors across the meta-community suggest that the remoteness from human settlements play an important role in shaping the mammalian communities in our study area in spite of their marked heterogeneity in species composition and habitat type. A coordinated and shared conservation strategy may therefore be effective to protect these communities. Among these we recommend monitoring the zone of interaction [21], as a framework to delineate the coupled human-natural system and hence facilitate the future assessment of the impact of human activities on biodiversity.

## Supporting information

S1 Appendix Model code. (TXT)

S1 Table Species checklist. List of mammal species and daily events recorded during camera trap surveys in five areas in the Udzungwa Mountains, Tanzania. Species are grouped by their taxonomic order and, within this, ordered by decreasing body mass.
(PDF)

S1 Fig Model results. Marginal posterior distributions with percentile-based 95% BCIs (dashed lines) for the parameters body mass, distance to the nearest river and distance to human settlements evaluated on occupancy (a), and for the parameters body mass and distance to human settlements on detection probability (b), from a multi-region hierarchical model applied to a meta-community of mammals in the Udzungwa Mountains of Tanzania.
(PDF)

S2 Fig Species-specific responses of occupancy and detection to covariates. Comparison between meta-community and species specific responses of occupancy to distance to the nearest river (a) and distance to human settlements (b), and of detection to distance to human settlements (c), from a multi-region hierarchical model applied to mammal communities across five surveyed areas in the Udzungwa Mountains of Tanzania. Red lines show posterior mean and 95% CRIs of the community mean hyperparameter, whereas species 95% CRIs that do not overlap zero are in blue.
(PDF)
