# Supplementary material for: A landscape-scale assessment of tropical mammals reveals the effects of habitat and anthropogenic disturbance on community occupancy
Source: PLoS One. 2019 Apr 19;14(4):e0215682. doi: 10.1371/journal.pone.0215682 (PMC6474625; doi:10.1371/journal.pone.0215682)
Supplement: S1 Table — List of mammal species and daily events recorded during camera trap surveys in five areas in the Udzungwa Mountains, Tanzania. Species are grouped by their taxonomic order and, within this, ordered by decreasing body mass. (PDF) [file pone.0215682.s002.pdf]

Order	Latin name	Body mass (Kg)	Matundu	Mbatwa	Lumemo	Ndundulu	Mwanihana	Total events
Afrotheria (n=6)								
1	*Loxodonta africana*	3940.043	42	56	4	10	18	130
2	*Orycteropus afer*	52.350	20	6	1	7	1	35
3	*Dendrohyrax validus*	2.430	1				9	10
4	*Rhynchocyon udzungwensis*	0.750				15	33	48
5	*Rhynchocyon cirnei*	0.490	15		10		2	27
6	*Petrodromus tetradactylus*	0.188	38					38
Artiodactyla (n=15)								
7	*Hippopotamus amphibius*	1417.490	15	2				17
8	*Syncerus caffer*	580.003	6	7	23	15	13	64
9	*Hippotragus niger*	227.499		1				1
10	*Tragelaphus strepiceros*	213.501		37				37
11	*Kobus ellipsiprymnus*	210.000	3		5			8
12	*Potamochoerus larvatus*	97.500	89	14	30	37	45	215

13	*Phacochoerus africanus*	82.500		6	2			8
14	*Cephalophus spadix*	56.000	9			23	17	49
15	*Tragelaphus scriptus*	43.250	31	104	60	28	49	272
16	*Sylvicapra grimmia*	19.500		2	8			10
17	*Cephalophus harveyi*	14.500	469	117	68	73	175	902
18	*Oreotragus oreotragus*	13.000		7				7
19	*Neotragus moschatus*	6.500	97	6	24	27	35	189
20	*Philantomba monticola*	6.250	2			37		39
21	*Madoqua kirkii thomasii*	5.300		72				72
Carnivora (n=18)								
22	*Crocuta crocuta*	63.000	51	9	48	6		114
23	*Panthera pardus*	55.000	58	22	51	33	14	178
24	*Aonyx capensis*	19.000				1		1
25	*Caracal caracal*	13.750		1				1
26	*Civettictis civetta*	12.000		34	2			36
27	*Leptailurus serval*	12.000		2	1			3
28	*Mellivora capensis*	9.000	18	15	3	9	5	50

29	*Ichneumia albicauda*	3.500		47				47
30	*Atilax paludinosus*	3.300	4	1	2	5	4	16
31	*Rhynchogale melleri*	2.500		1				1
32	*Genetta maculata*	2.225	48		27	2	2	79
33	*Genetta genetta*	2.000		62				62
34	*Nandinia binotata*	2.000			1	1	1	3
35	*Mungos mungo*	1.925	2	2				4
36	*Bdeogale crassicauda*	1.500	200	9	24	23	77	333
37	*Genetta servalina lowei*	1.055				28	14	42
38	*Galerella sanguinea*	0.550	2	3		1		6
39	*Helogale parvula*	0.300	15	1				16
Pholidota (n=1)								
40	*Smutsia temmninchii*	7.180		2				2
Primates (n=4)								
41	*Papio cynocephalus*	18.400	17	104	26		6	153
42	*Cercocebus sanjei*	8.000					43	43
43	*Cercopithecus mitis*	5.000	18	5	2	6	10	41

44	*Chlorocebus pygerythrus*	4.155		20				20
Rodentia (n=4)								
45	*Hystrix cristata*	20.000	20	71	33	6	8	138
46	*Thryonomys swinderianus*	4.060	1			4		5
47	*Cricetomys gambianus*	1.285	27		22	28	47	124
48	*Paraxerus vexillarius*	0.675	11			6	12	29
