# Supplementary figures and images for: A landscape-scale assessment of tropical mammals reveals the effects of habitat and anthropogenic disturbance on community occupancy
Source: PLoS One. 2019 Apr 19;14(4):e0215682. doi: 10.1371/journal.pone.0215682 (PMC6474625; doi:10.1371/journal.pone.0215682)

(a)

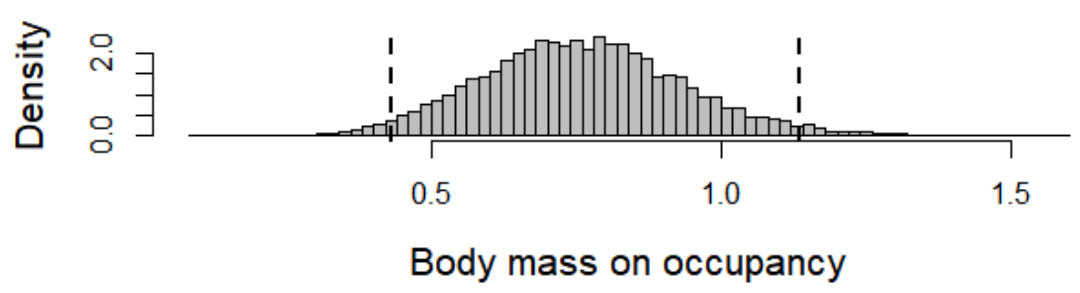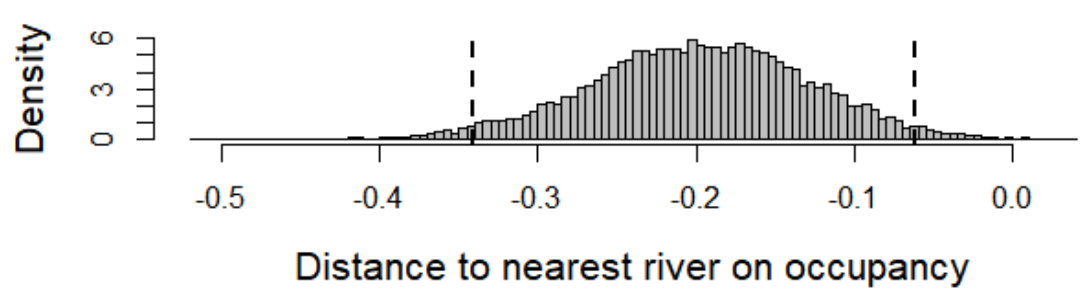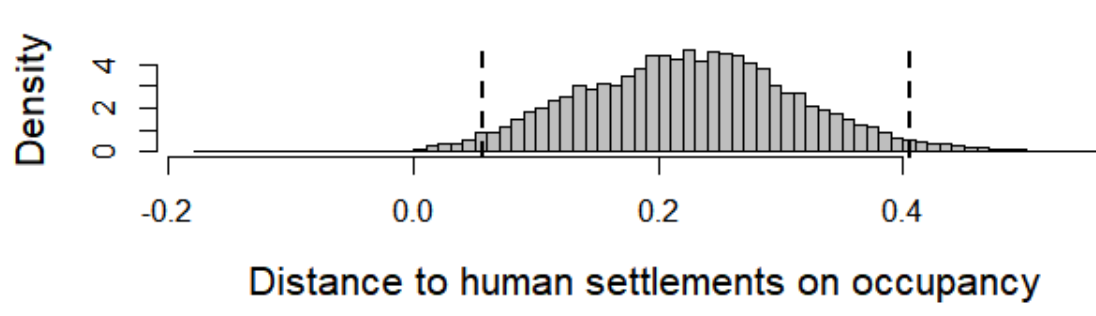

(b)

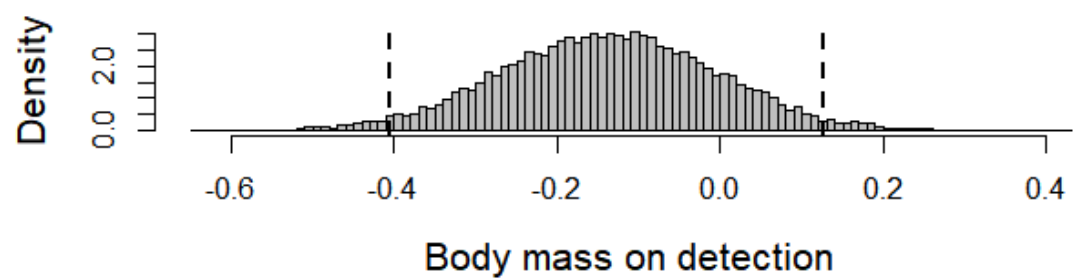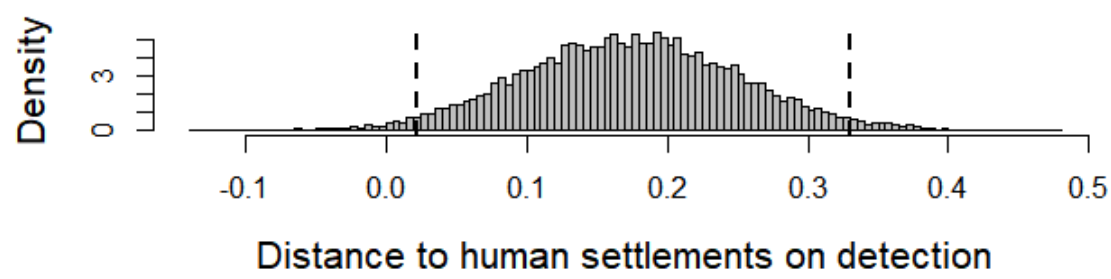

Supplement: S1 Fig — Marginal posterior distributions with percentile-based 95% BCIs (dashed lines) for the parameters body mass, distance to the nearest river and distance to human settlements evaluated on occupancy (a), and for the parameters body mass and distance to human settlements on detection probability (b), from a multi-region hierarchical model applied to a meta-community of mammals in the Udzungwa Mountains of Tanzania. (PDF) [file pone.0215682.s003.pdf]
