# Supplementary material for: A landscape-scale assessment of tropical mammals reveals the effects of habitat and anthropogenic disturbance on community occupancy
Source: PLoS One. 2019 Apr 19;14(4):e0215682. doi: 10.1371/journal.pone.0215682 (PMC6474625; doi:10.1371/journal.pone.0215682)
Supplement: S2 Fig — Comparison between meta-community and species specific responses of occupancy to distance to the nearest river (a) and distance to human settlements (b), and of detection to distance to human settlements (c), from a multi-region hierarchical model applied to mammal communities across five surveyed areas in the Udzungwa Mountains of Tanzania. Red lines show posterior mean and 95% CRIs of the community mean hyperparameter, whereas species 95% CRIs that do not overlap zero are in blue. (PDF) [file pone.0215682.s004.pdf]

(a)

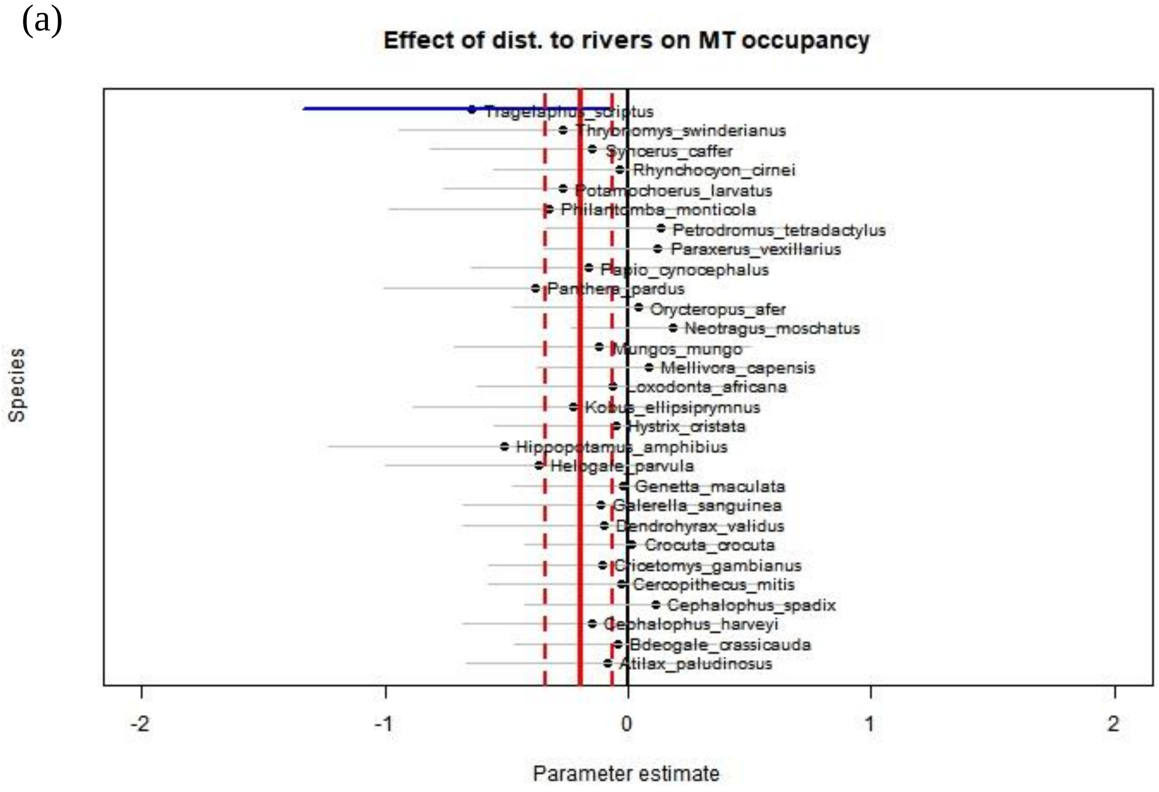
Effect of dist. to rivers on MT occupancy
Species
Tragelaphus_scriptus
Thryonomys_swinderianus
Syncerus_caffer
Rhynchocyon_cirnei
Potamochoerus_larvatus
Philantomba_monticola
Petrodromus_tetradactylus
Paraxerus_vexillarius
Papio_cynocephalus
Panthera_pardus
Orycteropus_afer
Neotragus_moschatus
Mungos_mungo
Mellivora_capensis
Loxodonta_africana
Kobus_ellipsiprymnus
Hystrix_cristata
Hippopotamus_amphibius
Helogale_parvula
Genetta_maculata
Galerella_sanguinea
Dendrohyrax_validus
Crocuta_crocuta
Cricetomys_gambianus
Cercopithecus_mitis
Cephalophus_spadix
Cephalophus_harveyi
Bdeogale_crassicauda
Atilax_paludinosus
-2
-1
0
1
2
Parameter estimate

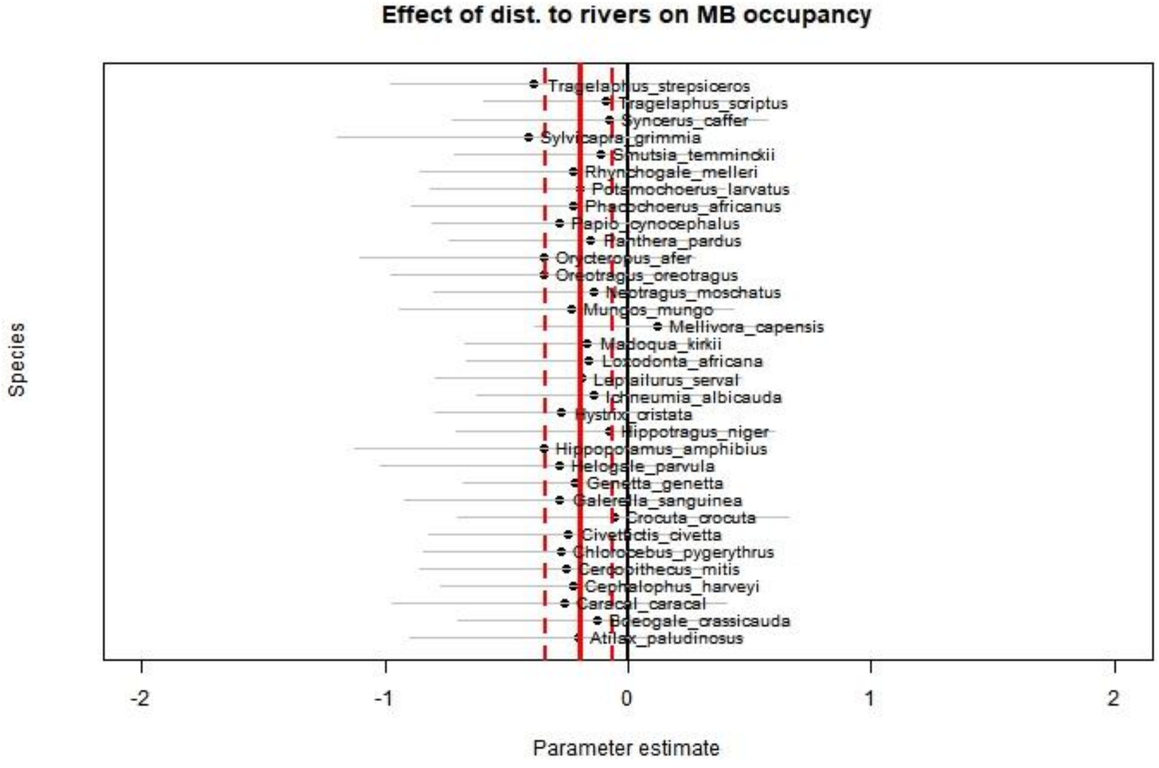
Effect of dist. to rivers on MB occupancy
Species
Tragelaphus_strepsiceros
Tragelaphus_scriptus
Syncerus_caffer
Sylvicapra_grimmia
Smutsia_temminckii
Rhynchogale_melleri
Potamochoerus_larvatus
Phacochoerus_africanus
Papio_cynocephalus
Panthera_pardus
Orycteropus_afer
Oreotragus_oreotragus
Neotragus_moschatus
Mungos_mungo
Mellivora_capensis
Madoqua_kirkii
Loxodonta_africana
Leptailurus_serval
Ichneumia_albicauda
Hystrix_cristata
Hippotragus_niger
Hippopotamus_amphibius
Helogale_parvula
Genetta_genetta
Galerella_sanguinea
Crocuta_crocuta
Civettictis_civetta
Chlorocebus_pygerythrus
Cercopithecus_mitis
Cephalophus_harveyi
Caracal_caracal
Bdeogale_crassicauda
Atilax_paludinosus
-2
-1
0
1
2
Parameter estimate

Effect of dist. to rivers on LU occupancy

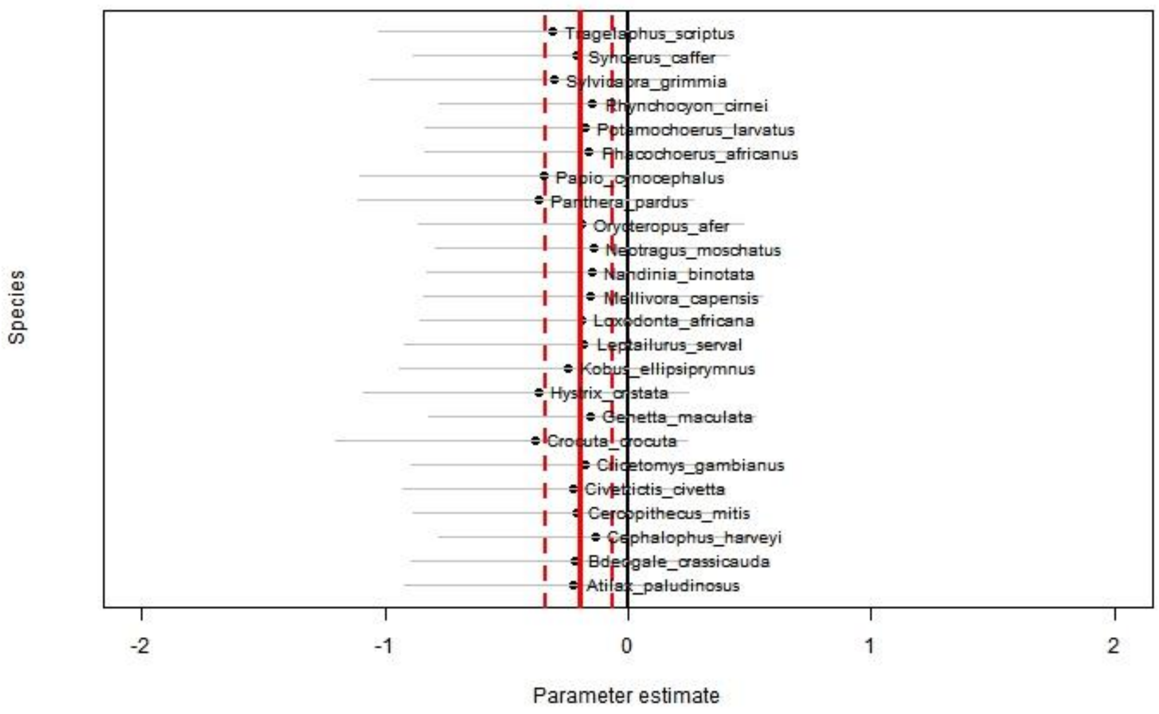

Effect of dist. to rivers on ND occupancy

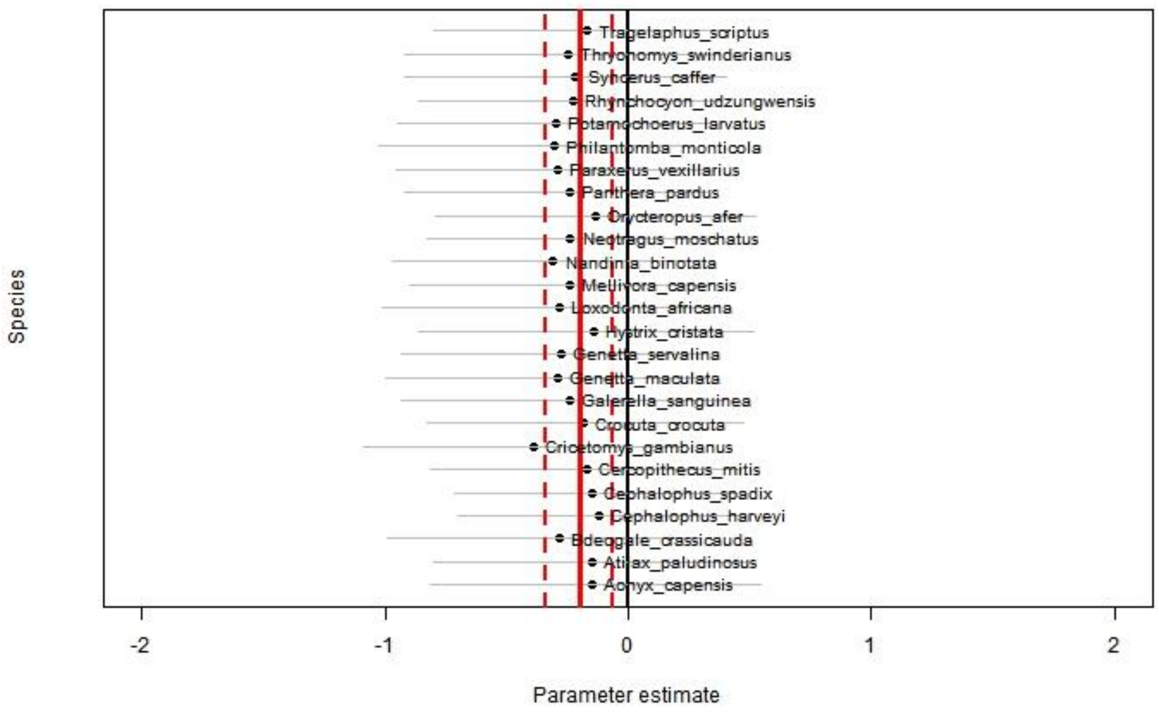

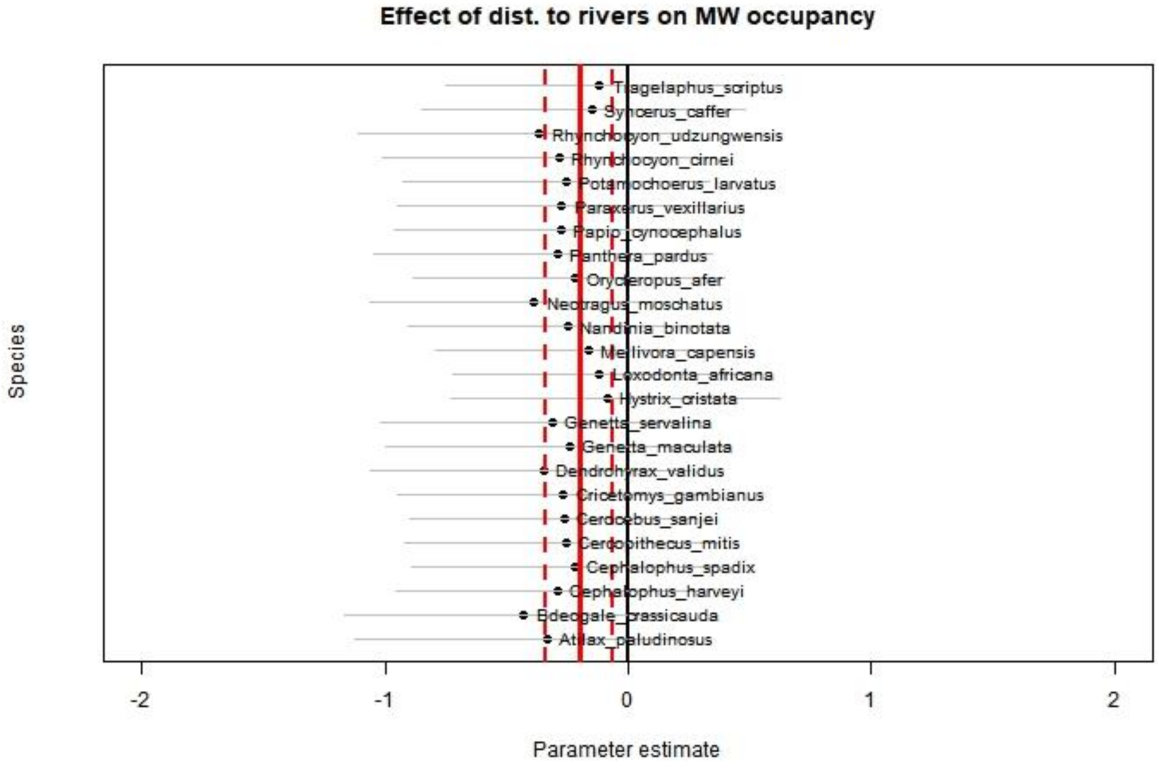
Effect of dist. to rivers on MW occupancy
Tragelaphus_scriptus
Syncerus_caffer
Rhynchocyon_udzungwensis
Rhynchocyon_cirnei
Potamochoerus_larvatus
Paraxerus_vexillarius
Papio_cynocephalus
Panthera_pardus
Orycteropus_afer
Neotragus_moschatus
Nandinia_binotata
Mellivora_capensis
Loxodonta_africana
Hystrix_cristata
Genetta_servalina
Genetta_maculata
Dendrohyrax_validus
Cricetomys_gambianus
Cercocebus_sanjei
Cercopithecus_mitis
Cephalophus_spadix
Cephalophus_harveyi
Bdeogale_crassicauda
Atilax_paludinosus
Species
-2
-1
0
1
2
Parameter estimate

(b)

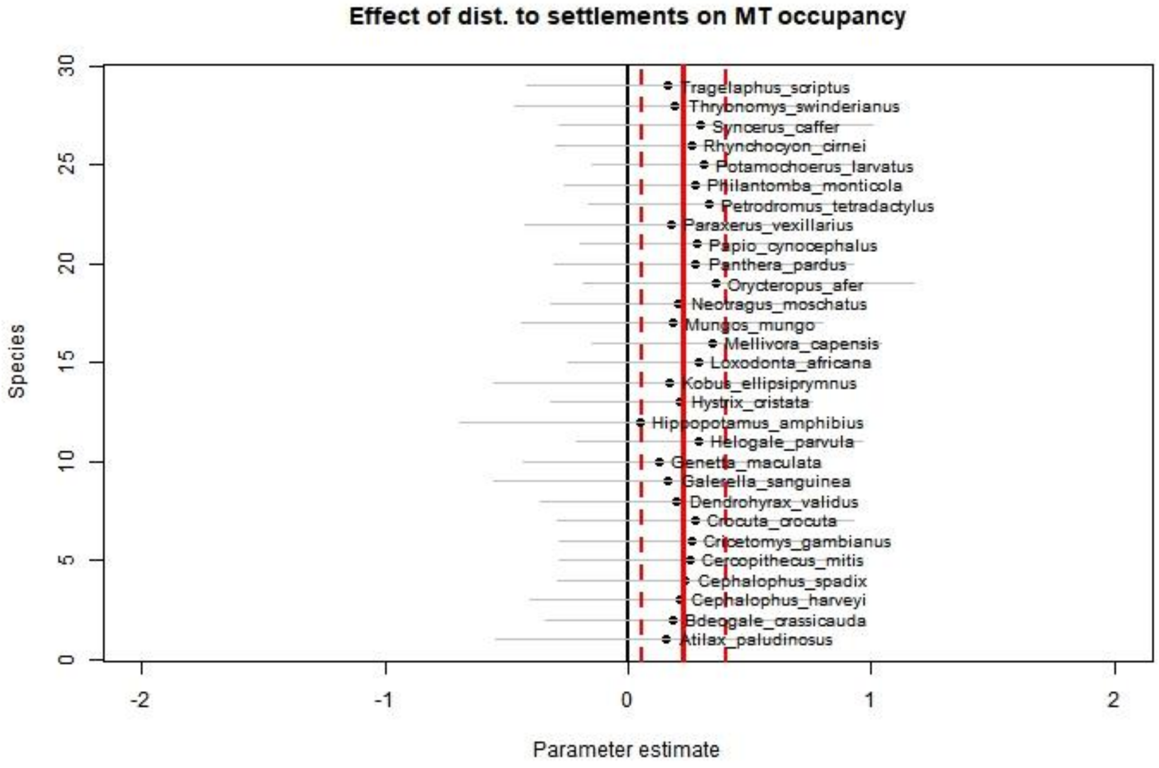
Effect of dist. to settlements on MT occupancy
Tragelaphus_scriptus
Thryonomys_swinderianus
Syncerus_caffer
Rhynchocyon_cirnei
Potamochoerus_larvatus
Philantomba_monticola
Petrodromus_tetradactylus
Paraxerus_vexillarius
Papio_cynocephalus
Panthera_pardus
Orycteropus_afer
Neotragus_moschatus
Mungos_mungo
Mellivora_capensis
Loxodonta_africana
Kobus_ellipsiprymnus
Hystrix_cristata
Hippopotamus_amphibius
Helogale_parvula
Genetta_maculata
Galerella_sanguinea
Dendrohyrax_validus
Crocuta_crocuta
Cricetomys_gambianus
Cercopithecus_mitis
Cephalophus_spadix
Cephalophus_harveyi
Bdeogale_crassicauda
Atilax_paludinosus
Species
0
5
10
15
20
25
30
-2
-1
0
1
2
Parameter estimate

Effect of dist. to settlements on MB occupancy

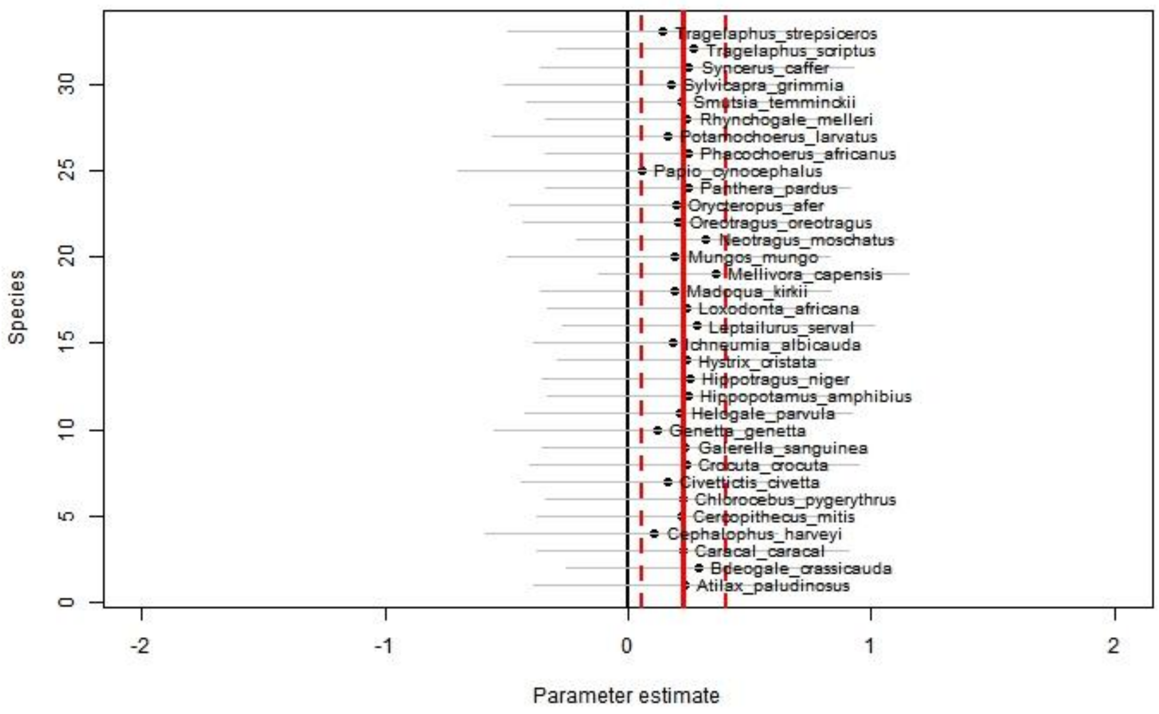

Effect of dist. to settlements on LU occupancy

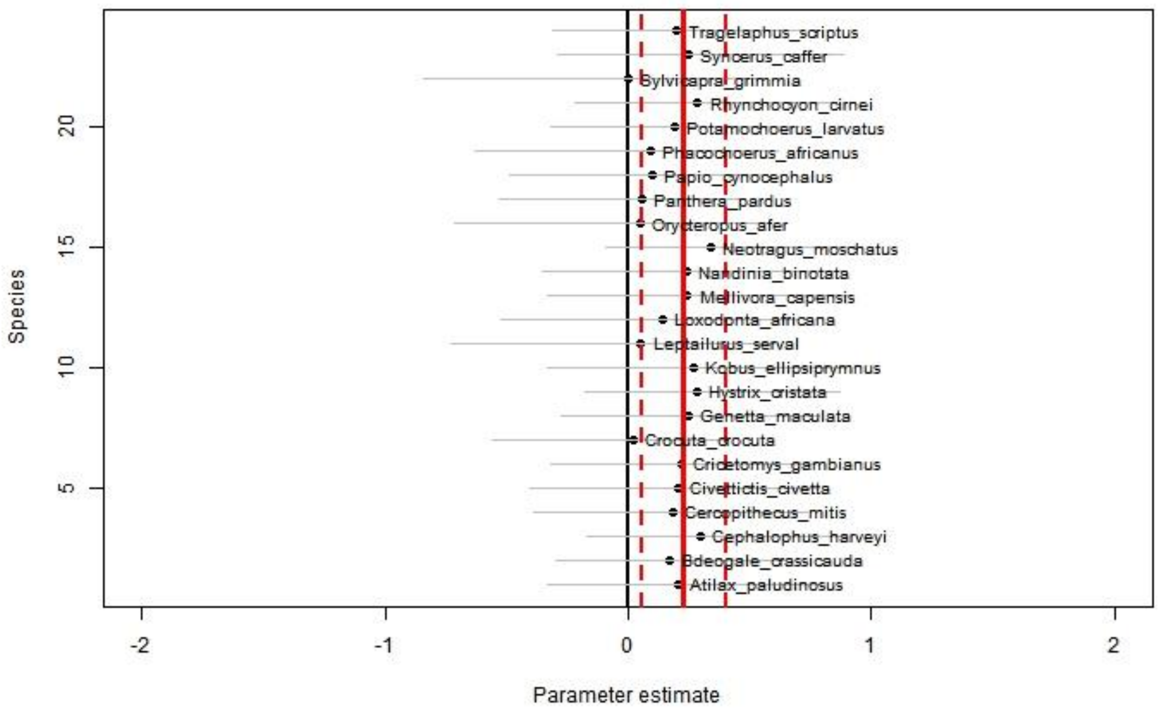

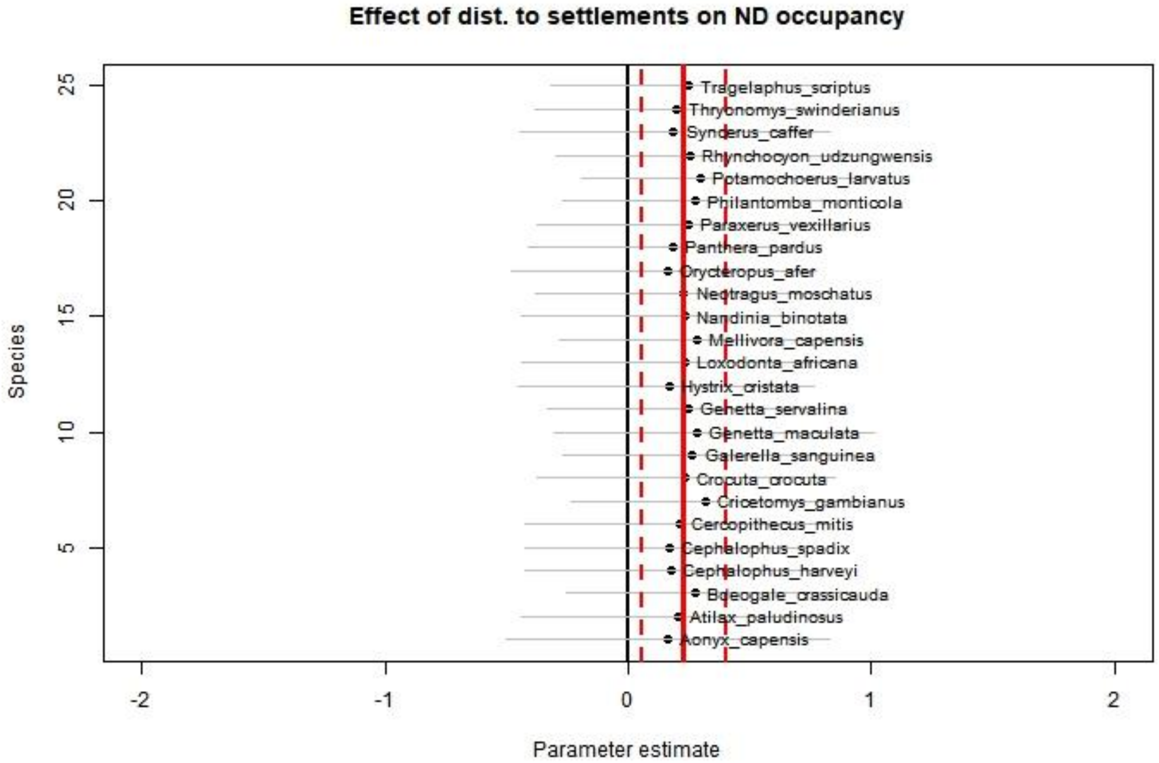
Effect of dist. to settlements on ND occupancy
Tragelaphus_scriptus
Thryonomys_swinderianus
Synderus_caffer
Rhynchocyon_udzungwensis
Potamochoerus_larvatus
Philantomba_monticola
Paraxerus_vexillarius
Panthera_pardus
Orycteropus_afer
Neotragus_moschatus
Nandinia_binotata
Mellivora_capensis
Loxodonta_africana
Hystrix_cristata
Genetta_servalina
Genetta_maculata
Galerella_sanguinea
Crocuta_crocuta
Cricetomys_gambianus
Cercopithecus_mitis
Cephalophus_spadix
Cephalophus_harveyi
Bdeogale_crassicauda
Atilax_paludinosus
Aonyx_capensis
Species
Parameter estimate
25
20
15
10
5
-2
-1
0
1
2

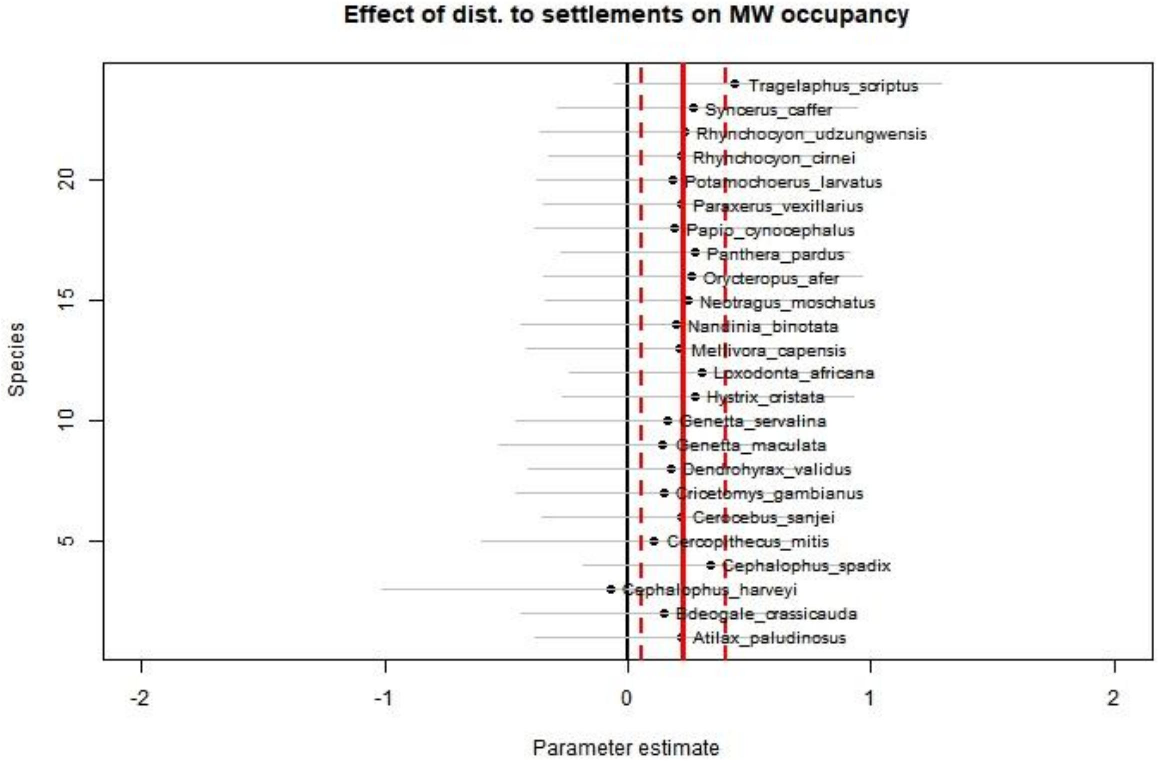
Effect of dist. to settlements on MW occupancy
Tragelaphus_scriptus
Syncerus_caffer
Rhynchocyon_udzungwensis
Rhynchocyon_cirnei
Potamochoerus_larvatus
Paraxerus_vexillarius
Papio_cynocephalus
Panthera_pardus
Orycteropus_afer
Neotragus_moschatus
Nandinia_binotata
Mellivora_capensis
Loxodonta_africana
Hystrix_cristata
Genetta_servalina
Genetta_maculata
Dendrohyrax_validus
Cricetomys_gambianus
Cercocebus_sanjei
Cercopithecus_mitis
Cephalophus_spadix
Cephalophus_harveyi
Bdeogale_crassicauda
Atilax_paludinosus
Species
Parameter estimate
20
15
10
5
-2
-1
0
1
2

(c)

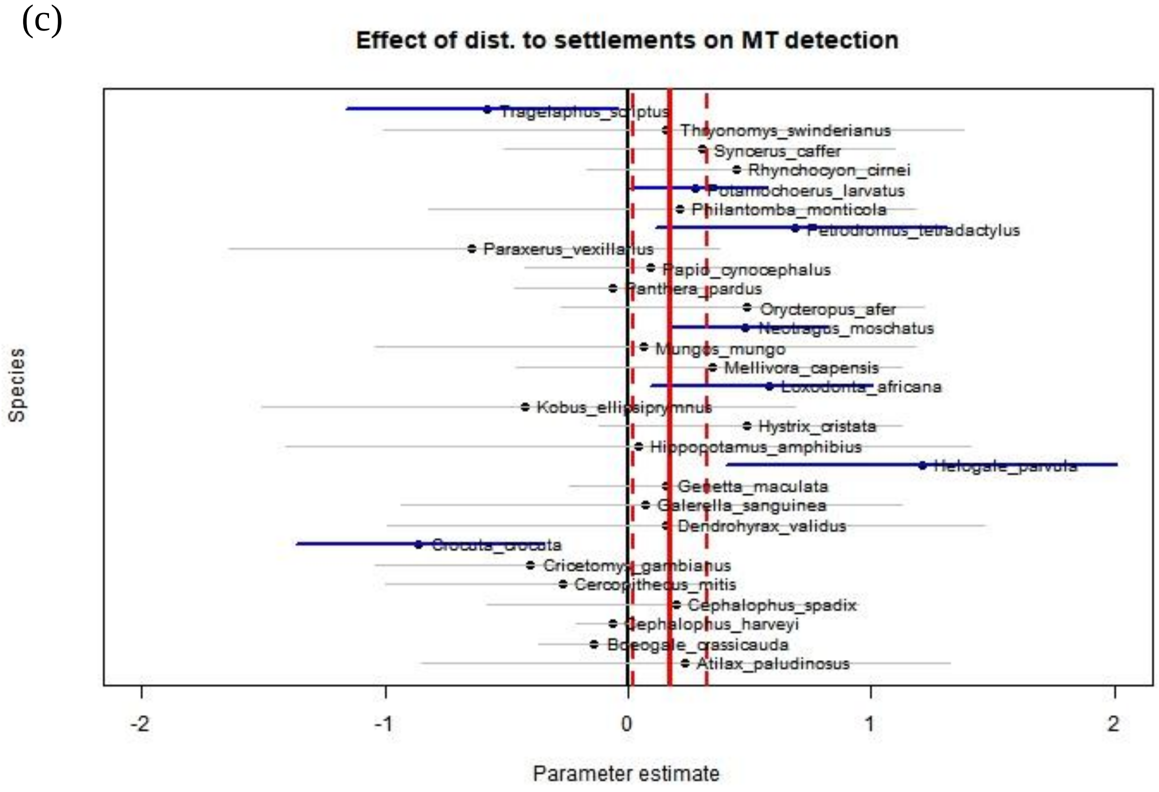
Effect of dist. to settlements on MT detection
Tragelaphus_scriptus
Thryonomys_swinderianus
Syncerus_caffer
Rhynchocyon_cirnei
Potamochoerus_larvatus
Philantomba_monticola
Petrodromus_tetradactylus
Paraxerus_vexillarius
Papio_cynocephalus
Panthera_pardus
Orycteropus_afer
Neotragus_moschatus
Mungos_mungo
Mellivora_capensis
Loxodonta_africana
Kobus_ellipsiprymnus
Hystrix_cristata
Hippopotamus_amphibius
Helogale_parvula
Genetta_maculata
Galerella_sanguinea
Dendrohyrax_validus
Crocuta_crocuta
Cricetomys_gambianus
Cercopithecus_mitis
Cephalophus_spadix
Cephalophus_harveyi
Bdeogale_crassicauda
Atilax_paludinosus
Species
-2
-1
0
1
2
Parameter estimate

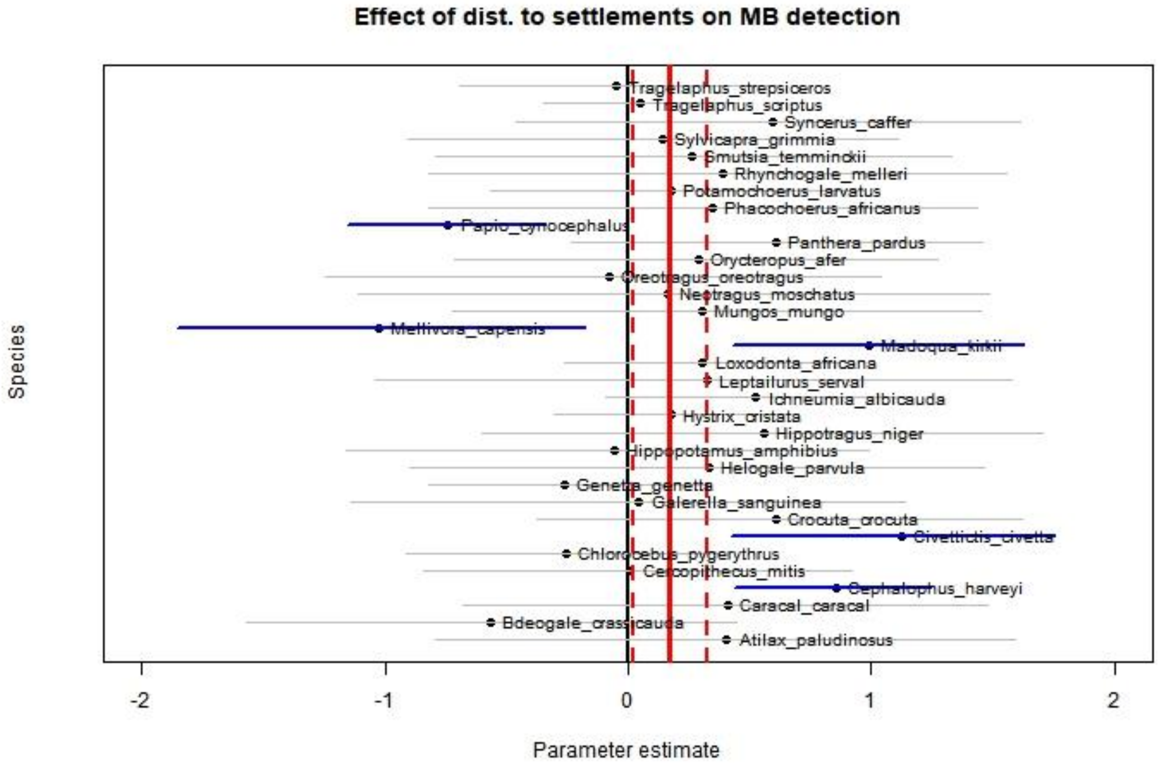
Effect of dist. to settlements on MB detection
Tragelaphus_strepsiceros
Tragelaphus_scriptus
Syncerus_caffer
Sylvicapra_grimmia
Smutsia_temminckii
Rhynchogale_melleri
Potamochoerus_larvatus
Phacochoerus_africanus
Papio_cynocephalus
Panthera_pardus
Orycteropus_afer
Oreotragus_oreotragus
Neotragus_moschatus
Mungos_mungo
Mellivora_capensis
Madoqua_kirkii
Loxodonta_africana
Leptailurus_serval
Ichneumia_albicauda
Hystrix_cristata
Hippotragus_niger
Hippopotamus_amphibius
Helogale_parvula
Genetta_genetta
Galerella_sanguinea
Crocuta_crocuta
Civettictis_civetta
Chlorocebus_pygerythrus
Cercopithecus_mitis
Cephalophus_harveyi
Caracal_caracal
Bdeogale_crassicauda
Atilax_paludinosus
Species
-2
-1
0
1
2
Parameter estimate

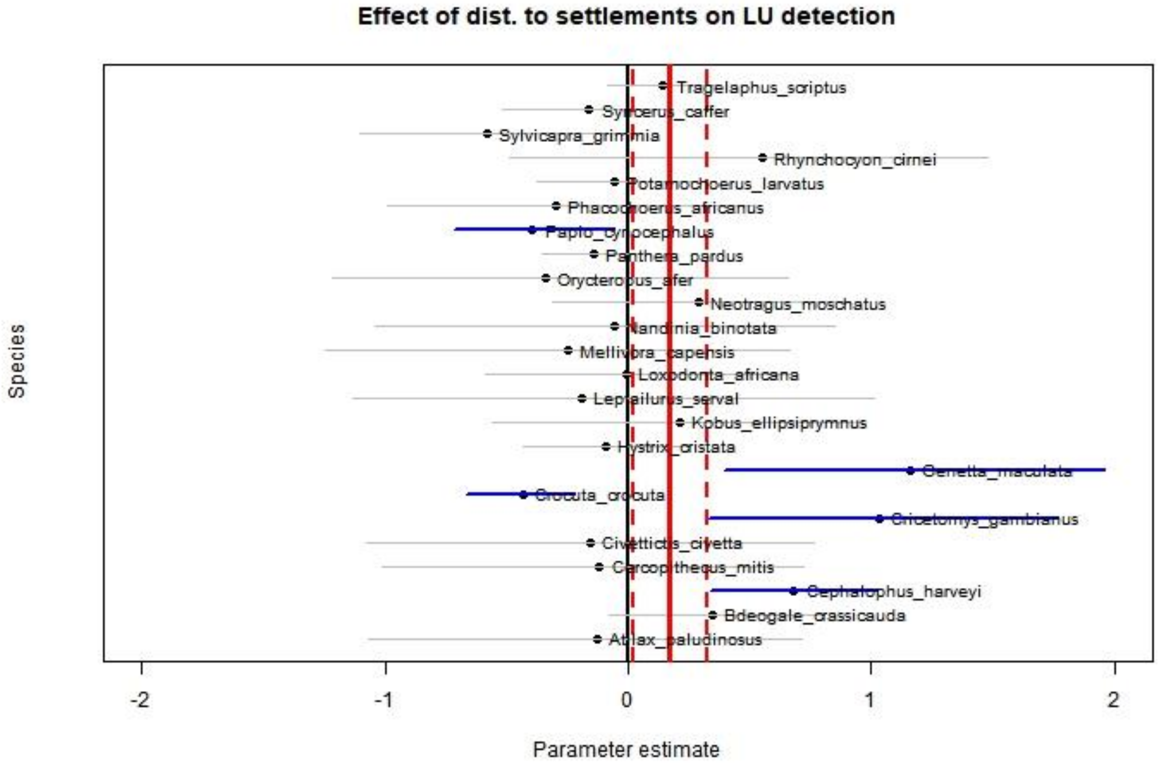
Effect of dist. to settlements on LU detection
Tragelaphus_scriptus
Syncerus_caffer
Sylvicapra_grimmia
Rhynchocyon_cirnei
Potamochoerus_larvatus
Phacochoerus_africanus
Papio_cynocephalus
Panthera_pardus
Orycteropus_afer
Neotragus_moschatus
Nandinia_binotata
Mellivora_capensis
Loxodonta_africana
Leptailurus_serval
Kobus_ellipsiprymnus
Hystrix_cristata
Genetta_maculata
Crocuta_crocuta
Cricetomys_gambianus
Civettictis_civetta
Cercopithecus_mitis
Cephalophus_harveyi
Bdeogale_crassicauda
Atilax_paludinosus
Species
-2
-1
0
1
2
Parameter estimate

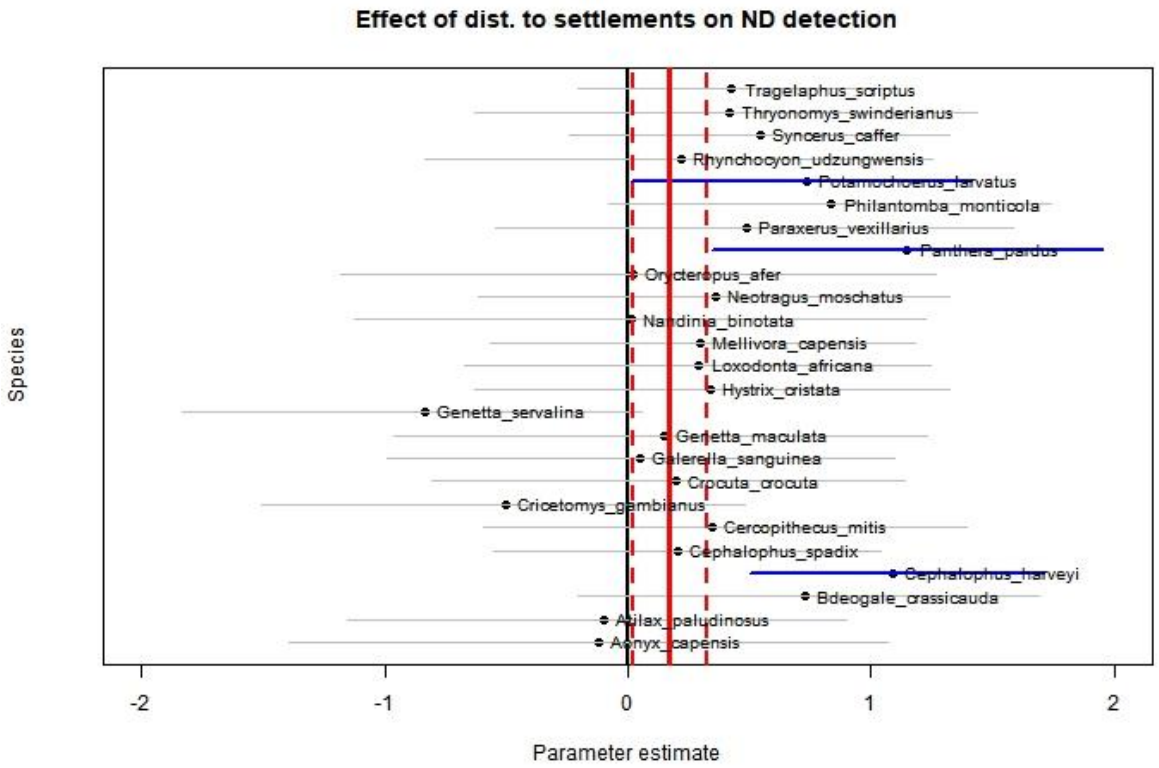
Effect of dist. to settlements on ND detection
Tragelaphus_scriptus
Thryonomys_swinderianus
Syncerus_caffer
Rhynchocyon_udzungwensis
Potamochoerus_larvatus
Philantomba_monticola
Paraxerus_vexillarius
Panthera_pardus
Orycteropus_afer
Neotragus_moschatus
Nandinia_binotata
Mellivora_capensis
Loxodonta_africana
Hystrix_cristata
Genetta_servalina
Genetta_maculata
Galerella_sanguinea
Crocuta_crocuta
Cricetomys_gambianus
Cercopithecus_mitis
Cephalophus_spadix
Cephalophus_harveyi
Bdeogale_crassicauda
Atilax_paludinosus
Aonyx_capensis
Species
-2
-1
0
1
2
Parameter estimate

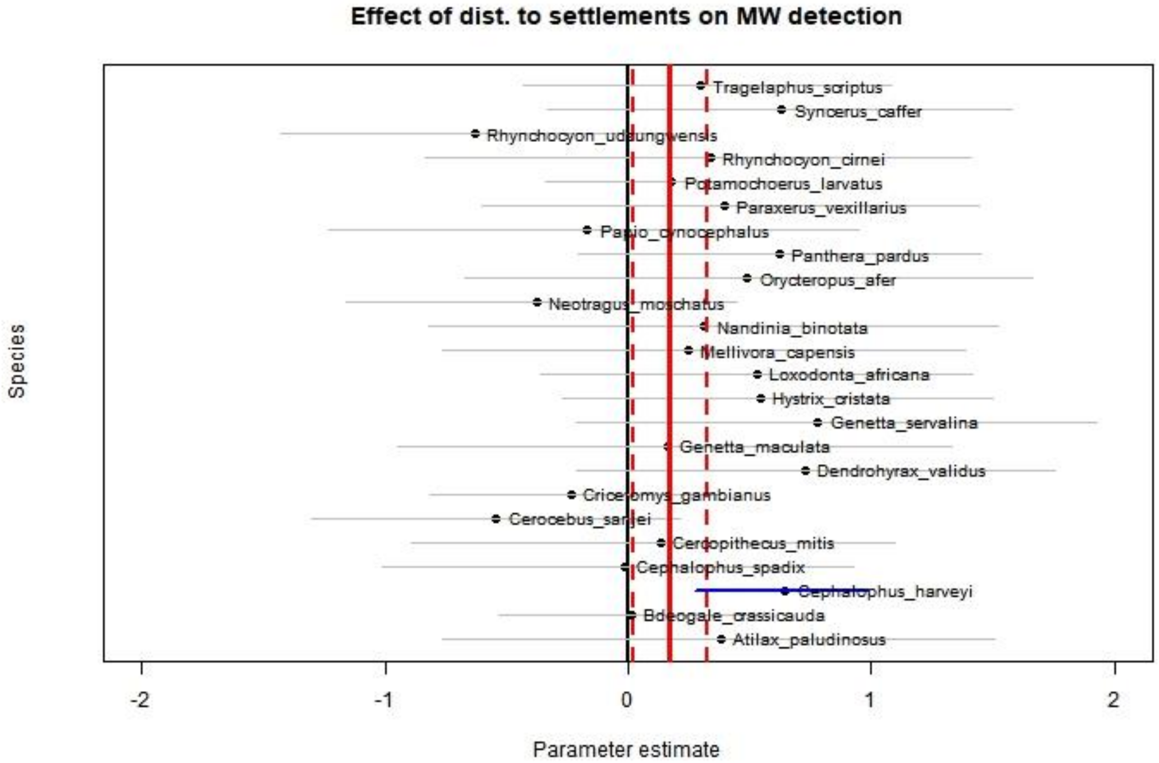

Effect of dist. to settlements on MW detection
Tragelaphus_scriptus
Syncerus_caffer
Rhynchocyon_udzungwensis
Rhynchocyon_cirnei
Potamochoerus_larvatus
Paraxerus_vexillarius
Papio_cynocephalus
Panthera_pardus
Orycteropus_afer
Neotragus_moschatus
Nandinia_binotata
Mellivora_capensis
Loxodonta_africana
Hystrix_cristata
Genetta_servalina
Genetta_maculata
Dendrohyrax_validus
Cricetomys_gambianus
Cerocebus_sanjei
Cercopithecus_mitis
Cephalophus_spadix
Cephalophus_harveyi
Bdeogale_crassicauda
Atilax_paludinosus
Species
-2
-1
0
1
2
Parameter estimate
